# Data on the generation of rabbit infections and RPR titre changes in serum samples from syphilis patients at follow-up

**DOI:** 10.1016/j.dib.2018.10.075

**Published:** 2018-11-07

**Authors:** Wen Liu, Xiaohong Zhang, Tie Zhao, Chenglong Zhou, Junxia Duan, Feijun Zhao

**Affiliations:** a Hunan Provincial Key Laboratory for Special Pathogens Prevention and Control, Hunan Province Cooperative Innovation Center for Molecular Target New Drug Study, Pathogentic Biology Institute, University of South China, Hengyang 421001, China; b Department of Histology and Embryology, Hengyang Medical College, University of South China, Hengyang 421001, China

**Keywords:** *Treponema pallidum*, Rabbit, RPR, ELISA

## Abstract

The data presented in this article are related to the research article entitled “Performance of novel infection phase-dependent antigens in syphilis serodiagnosis and treatment efficacy determination”. The rabbit model [1], [2] is an appropriate animal model for studying syphilis, a classic sexually transmitted disease (STD). Live *Treponema pallidum* (*T. pallidum*, Tp) and inactivated *T. pallidum* were inoculated in the backs of New Zealand rabbits. RT-PCR was performed to determine whether *T. pallidum* DNA could be detected in different groups. Sixty paired serum samples from patients at follow-up were tested by RPR and recombinant Tp0971-, Tp0768-, Tp0462- and Tp92-based ELISA.

**Specifications table**

**Table:** 

Subject area	Biology
More specific subject area	Microbiology, *Treponema pallidum (T. pallidum),* diagnosis
Type of data	Table, figure
How data was acquired	Microscope, camera, polymerase chain reaction
Data format	Raw data, analysed data
Experimental factors	Rabbits were infected with live *T. pallidum o*r inactive *T. pallidum*. Human serum samples were stored at −80 °C.
Experimental features	Data illustrated different antigen-antibody reactions between different groups (live *T. pallidum-* and inactive *T. pallidum*-inoculated rabbits)
Data source location	University of South China, Hunan, China
Data accessibility	The data are available with this article
Related research article	The data presented herein is related to the research article entitled "Performance of novel infection phase-dependent antigens in syphilis serodiagnosis and treatment efficacy determination." (Liu W, Deng M, Zhang X, Yin W, Zhao T, Zeng T, Liu S, Xiao Y, Zhang L, Luo X, Zhao F., Clin Chim Acta. 2018 Oct 13. pii: S0009–8981(18)30543-6. doi: 10.1016/j.cca.2018.10.017. [Epub ahead of print] PMID:30326217.

**Value of the data**

• The rabbit infection model, a useful model for studying *Treponema pallidum* [1], [2], can provide data after the inoculation of live T*. pallidum* and inactivated T*. pallidum* in the backs of New Zealand rabbits to observe specific antigen-antibody reactions.
• The data showed the relationships between the RPR titre change and ELISA in 60 paired follow-up samples from syphilis patients.
• The data provide new ideas for syphilis diagnosis.

## 1 Data

**Fig. 1:**
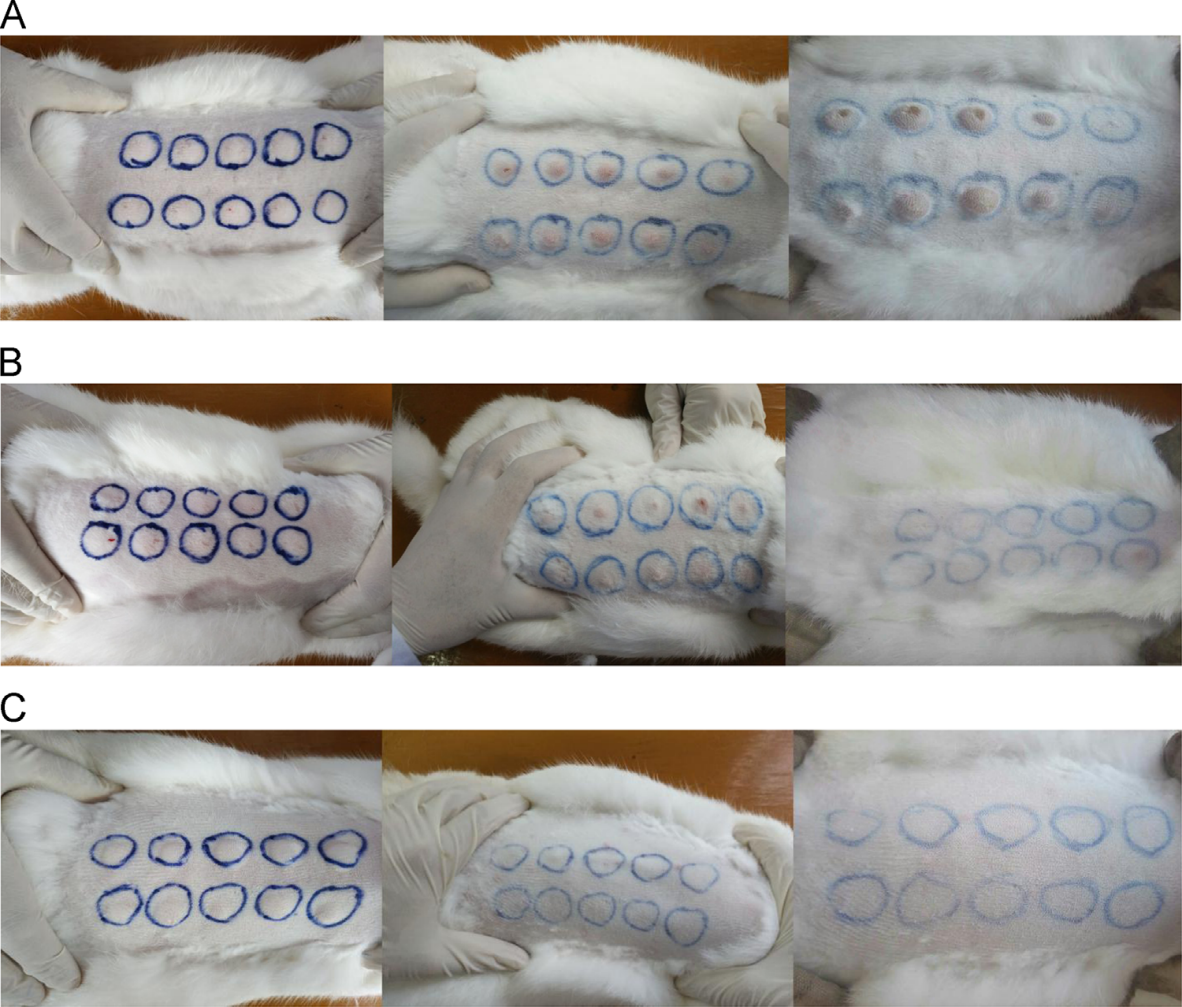
Representative images of rabbits after intradermal challenge with the *T. pallidum* Nichols strain at eight locations on the back. A. The shaved back of a New Zealand rabbit in the live Tp group on day 1, day 12, and day 24, from left to right, B. The shaved back of a New Zealand rabbit in the inactivated Tp group on day 1, day 12, and day 24, from left to right, C. The shaved back of a New Zealand rabbit in the negative control group on day 1, day 12, and day 24, from left to right.

The data presented in this article are mainly related to the generation of rabbit infections and interesting findings concerning the relationship between RPR titre changes and ELISA results in follow-up samples from syphilis patients. When infected with Tp, different groups of New Zealand rabbits (live Tp group, inactivated Tp group and negative control group) presented different results (Fig. 1). Sixty paired follow-up samples of human serum (patients hospitalized in the Regional Affiliated Hospitals of the University of South China between September 2014 and September 2016) were tested by RPR (qualitative assay) and Tp0971-, Tp0768-, Tp0462- and Tp92-based ELISA. The relationships between the RPR titre change and the OD450 nm of each recombinant protein-based ELISA are shown in Table 1.

**Table 1:** Relationship between the RPR titre change and the ΔOD450 nm from recombinant protein-based ELISA in 60 paired serum samples from patients at follow-up.

Sample	RPR titre decrease	ΔTp0971(OD450 nm)	ΔTp0768(OD450 nm)	ΔTp0462(OD450 nm)	ΔTp92(OD450 nm)
1	2	0.3949	−0.0445	0.0266	−0.0987
2	2	0.3814	0.4756	0.2942	0.2588
3	1	−0.0037	0.0174	0.2098	0.1893
4	2	0.5564	0.3922	0.4759	0.4164
5	1	0.1397	1.4729	0.0204	0.2578
6	2	0.5492	0.3734	0.7591	0.2892
7	4	1.3151	0.5152	−0.1305	0.2611
8	4	0.6454	0.7085	0.1162	0.0816
9	4	1.0972	0.7236	0.1982	0.0632
10	1	0.2246	0.0321	0.1138	0.1309
11	1	0.1015	0.1574	−0.0577	0.1804
12	4	0.7721	0.8742	−0.3067	0.0174
13	4	0.8008	−0.0365	0.1757	0.1104
14	4	0.6171	0.5319	0.2091	0.1814
15	4	0.8401	0.6655	−0.0312	0.3186
16	4	1.1203	0.5956	−0.0869	0.0199
17	4	1.2202	0.2474	−0.2076	−0.0044
18	2	0.3800	0.6922	0.5903	0.0929
19	4	1.1004	1.1129	0.6764	0.3635
20	4	0.6102	0.2234	0.6131	0.0965
21	2	0.4511	0.3252	1.1353	0.1761
22	4	0.6122	0.6885	0.0023	0.0012
23	4	0.5734	0.1936	−0.1664	0.0903
24	4	0.6759	−0.0693	−0.1006	−0.0668
25	4	0.7590	0.7944	0.0489	−0.0202
26	2	0.4164	0.4562	0.2694	0.1368
27	1	0.2902	−0.0155	0.1805	0.0550
28	2	0.7922	0.4509	−0.2007	0.0686
29	2	0.4082	0.4066	0.0313	0.1663
30	4	0.5754	0.4340	0.8414	0.0203
31	1	0.3719	0.0998	0.0732	0.1416
32	1	0.3471	0.4659	0.1830	−0.0185
33	1	0.3513	0.3204	−0.0134	0.0263
34	1	0.2218	0.2190	0.0992	0.1410
35	1	0.1364	−0.2205	−0.0266	−0.2737
36	1	0.2218	0.2229	0.1960	0.1130
37	1	0.2116	0.2104	0.0691	0.0429
38	1	−0.0029	0.0244	−0.0230	0.0727
39	1	0.0514	0.0534	0.1202	0.3632
40	2	0.6902	−0.5256	0.1025	0.1646
41	1	0.5210	0.4334	0.1130	0.2461
42	4	0.4503	0.4252	1.1353	0.3712
43	1	0.2122	0.2186	0.7010	0.1603
44	4	0.5734	0.7036	0.8336	0.0032
45	4	0.6759	0.6707	0.5994	0.0498
46	2	0.3590	0.3944	0.4489	0.1068
47	1	0.4164	0.0762	0.0694	0.1250
48	2	0.2807	0.2445	0.4805	−0.0614
49	2	0.2922	0.4509	0.3993	0.2363
50	4	0.4082	0.5066	0.7313	−0.2097
51	1	0.0754	0.034	0.1414	−0.3884
52	2	0.3719	0.4609	0.4730	0.0515
53	2	0.2471	0.2659	0.4303	0.0712
54	2	0.3513	0.4204	0.8866	0.2110
55	2	0.4098	0.3190	0.3992	0.0963
56	2	0.4054	0.2895	0.3697	0.1830
57	2	0.5168	0.1909	0.3960	0.1129
58	1	0.1166	0.4104	0.0690	0.1427
59	1	−0.0024	0.0244	−0.0230	0.4332
60	2	0.0517	0.5014	0.4202	0.2346

#ΔOD450 nm, the difference in OD450 nm values between the follow-up samples.

## 2 Experimental design, materials and methods

### 2.1 The generation of rabbit infections

The *T. pallidum* Nichols strain used in this study was a generous gift from Tianci Yang (Zhongshan Hospital, Medical College of Xiamen University, Xiamen, China) and was maintained in the Pathogenic Biology Institute, Medical College, University of South China. The resuscitation and proliferation of *T. pallidum* were performed as previously described [3]. Inactivated *T. pallidum* was the live *T. pallidum* strain treated with ultraviolet light for 30 min. Male New Zealand rabbits (*n* = 18, age 4–6 months, weight 2.5–3.0 kg, Department of Laboratory Animals, University of South China) were raised at 18–20 °C, with antibiotic-free food and water. The rabbits were randomly divided into 3 groups of 6 rabbits each, as follows: a live *T. pallidum*-inoculated group, an inactivated *T. pallidum*-inoculated group and an untreated group. Then, biopsy samples (at day 24 post-challenge) were analysed by RT-PCR (LightCycler® 96, Switzerland) to determine whether *T. pallidum* DNA could be detected in the different groups (Table 2).

**Table 2:** RT-PCR results for biopsy samples from different rabbit groups (at day 24 post-challenge).

Group	Mean Ct	SD	CV (%)
MMP-1#	19.64	0.32	1.63
Live *T. pallidum* group	24.82	0.63	2.54
Inactivated *T. pallidum* group	0	0	–
Negative control group	0	0	–

#Reference gene, GenBank accession number M17820.

– indicates that data are not available.

### 2.2 RPR titre changes and ΔOD450 nm values of recombinant protein-based ELISA for 60 paired human serum samples from patients at follow-up

RPR (qualitative assay) and recombinant protein-based ELISA were performed as described in the research article entitled “Performance of novel infection phase-dependent antigens in syphilis serodiagnosis and treatment efficacy determination”. The relationships are shown in Table 1.
