# Supplementary material for: Data on the generation of rabbit infections and RPR titre changes in serum samples from syphilis patients at follow-up
Source: Data Brief. 2018 Nov 7;21:2237–41. doi: 10.1016/j.dib.2018.10.075 (PMC6276544; doi:10.1016/j.dib.2018.10.075)
Supplement: Supplementary file 1 — Supplementary material. [file mmc1.pdf]

Authorship & Conflict of Interest Statement

To the editors:

We are submitting the data article “Data on the generation of rabbit infections and RPR titre changes in serum samples from syphilis patients at follow-up ” for consideration at《Clin Chim Acta》. We warrant that the manuscript represents original work that is not being considered for publication, in whole or in part, in another journal, book, conference proceedings, or government publication with a substantial circulation.

We warrant that all of the authors have contributed substantially to the manuscript and approved the final submission, and no person or persons other than the authors listed have contributed significantly in its preparation.

We warrant no conflicts of interest.

Sincerely,

Feijun Zhao

Institute of Pathogenic Biology and Key Laboratory of Special Pathogen Prevention and Control of Hunan Province, University of South China, No. 28 West Changsheng Road, Hengyang 421001, Hunan Province, P.R. China

Email: nhdxzhfj@163.com
